# Transitioning preclinical students into clerkships amidst curricular disruptions from the COVID-19 pandemic

**DOI:** 10.1080/10872981.2021.1996216

**Published:** 2021-10-28

**Authors:** Ernie L. Esquivel, Paolo De Angelis, John K. Chae, Joseph E. Safdieh, Erika L. Abramson, Yoon Kang

**Affiliations:** a Division of General Internal Medicine, Department of Medicine, Weill Cornell Medicine, New York, NY, USA; b School of Medicine, Weill Cornell Medicine, New York, Ny, USA; c Department of Neurology, Weill Cornell Medicine, New York, Ny, USA; d Department of Pediatrics, Weill Cornell Medicine, New York, NY, USA; e Department of Population Health Sciences, Weill Cornell Medicine, New York, Ny, USA

**Keywords:** Clinical skills, medical education research, collaborative/peer-to-peer teaching, curriculum planning

## Abstract

The COVID-19 pandemic resulted in significant disruptions to medical education. The patient care space was unavailable as a learning environment, which compounded the complexity of preparing students for clerkships with a traditional transition to clerkship (TTC) curriculum. We developed a multimodal, structured approach to re-introduce students to the clinical space prior to the start of clerkships. 105 second year medical students completed a 4-week clinical enhancement course. A modified Delphi method was used to select core topics, which were then anchored to key Entrustable Professional Activities (EPAs). Students participated in 9 virtual problem-based cases, workshops and multiple supervised patient encounters. Students were surveyed before, during, and after the course; responses were compared with paired t-tests. 25.9% rated the course as excellent, 44.2% as very good, and 19.5% as good. Compared to baseline, self-perceived efficacy grew significantly (P < 0.05) across all EPAs. Improvements in key competencies were sustained when students were surveyed 2 weeks into their first clerkship. This was a well-received, novel course, focused on helping students transition back into the clinical space through a multimodal teaching approach. This framework may be used by other institutions seeking to restructure their TTC initiatives.

## Introduction

The transition from the preclinical phase of the medical school curriculum to the clinical clerkships poses many challenges. In most medical schools, the preclinical curriculum places relatively less emphasis on teaching practical skills, such as history-taking, physical examination, oral presentations, documentation, and familiarity with team dynamics [1]. With limited time spent in the clinical environment, preclinical medical students typically report high anxiety and low confidence in their clinical skills [2–5]. Similarly, clerkship directors express concerns about students’ lack of preparedness [6,7]. Transition to clerkship courses (TTCs) have been developed to focus on teaching skills necessary to navigate the new learning spaces of the hospital and outpatient clinics; yet, studies have questioned the effectiveness of such initiatives [1].

During the past year, the existing problem of transitioning students was exacerbated by COVID-19, which caused significant disruptions in clinical care and medical education worldwide. Students were removed from rotations early on in the pandemic to minimize exposure to a deadly disease, especially with inadequate availability of personal protective equipment and limited testing capabilities. Teaching was moved to virtual learning platforms, but the lack of access to patients and the need for social distancing imposed a pause on the learning of clinical skills. These curricular interruptions compounded the already existing challenge of adequately preparing students for the clinical phase of training, and concerns about the downstream effects on clerkship performance and residency applications have already been voiced [8,9]. Various initiatives have been described in the literature to combat the pandemic’s impact on traditional medical education [10–16]; yet specific strategies to effectively transition students into the clerkship curriculum in the COVID-19 and post-COVID-19 eras are still lacking.

We developed a transitional course in response to COVID-19 disruptions aimed at facilitating the entry of medical students into the clinical phase of the curriculum while still operating under continued COVID-19 institutional limitations and policies. We relied on a multimodal approach targeted at enhancing self-efficacy in core clinical competencies while using near-peer mentors. The goals of this study were to: 1) detail the conceptual and structured approach to the development of the course, 2) describe the novel role of near-peer mentors and logistics of course implementation, and 3) report results from student feedback and self-efficacy surveys. This paper can serve as a guide for other institutions wishing to re-evaluate their own TTC courses or, more broadly, incorporate core tenets of our course in other areas of their curricula.

## Materials and methods

### Setting

This study was approved by the Institutional Review Board of Weill Cornell Medicine. (protocol # 21–02023322) under waiver of informed consent as an educational project. Throughout the Spring and Fall of 2020, in-person clinical activities for preclinical medical students were suspended; most traditional TTC exercises were cancelled, with the exceptions of some which were amenable to a virtual platform (e.g., student-to-student shadowing on rounds). The course was organized by two faculty members in collaboration with 4th year medical student volunteers. The course was designed for 2nd year medical students who, at our institution, transition from pre-clinical to clinical curriculum in January of their 2nd year.

### Course content selection

Focus groups were conducted with 2nd year students to identify areas of perceived lack of preparation. Two additional focus groups were conducted with senior students to explore how they could have been better prepared for clerkships. Learner needs were categorized into the domains of medical knowledge, clinical skills and attitudes and mapped to the Association of American Medical Colleges (AAMC) Core Entrustable Professional activities (EPAs) [17]. Organizers relied on a modified Delphi method to develop tangible learning objectives across domains; areas of weakness and core topics that had emerged in focus groups were prioritized based on relevance to clerkship preparation. Each organizer submitted an anonymous ranking list of topics to the course faculty leader, and content areas that were most frequently identified as important drove design of course activities.

### Course design

We utilized a multi-modal teaching approach; educational activities primarily consisted of modified problem-based learning (PBL) sessions, simulated patient encounters using virtual learning platforms, and in-person exercises conducted in accordance with COVID-19 restrictions. Supplemental lectures addressing core knowledge areas were also provided. There were seven in-person exercises: (1) three history and physical examination (H&P) sessions guided by student or faculty preceptors in the inpatient or outpatient settings, (2) a half-day shadowing exercise in the operating room (OR), (3) a half-day shadowing exercise during which students followed pre-rounds and rounds in the hospital, and (4) an interprofessional exercise (IPE) half-day with a nurse on an inpatient ward during which students observed nursing tasks and the interactions between physicians and nurses. We recruited 26 4th year medical students to lead small group session as well as 33 4th year medical students and 30 faculty members as H&P preceptors. For the IPE exercise, each student was paired with a nurse clinician.

**Figure 1.**
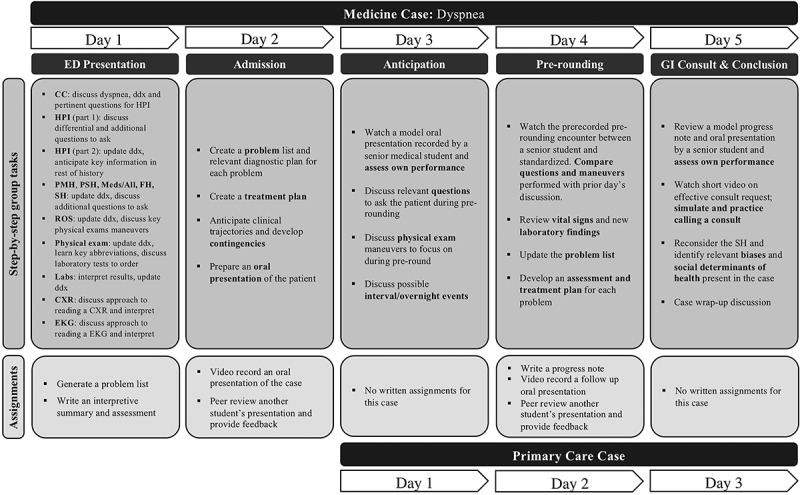
Example of a self-directed learning case with associated assignments. On day 3 of the medicine case, students also simultaneously started working on the primary care case. Abbreviations used: CC = chief complaint, CXR = chest x-ray, ED = emergency department, EKG = electrocardiogram, FH = family history, GI = gastrointestinal, HPI = history of present illness, Labs = laboratory, Meds/All = medications and allergies, PMH = past medical history, PSH = past surgical history, ROS = review of systems, SH = social history

We developed 9 modified PBL cases, each simulating an initial patient encounter on one of the required clerkships. The details of these cases and their associated EPAs are listed in Table 1, and an example case is shown in Figure 1. Teams of 4 students worked virtually and collaboratively using Google Forms (Google, Mountain View, CA), and were asked to reason through each case in a hypothesis-driven fashion, offering a differential diagnosis, requesting additional history, interpreting physical examination, laboratory and radiologic data as the case evolved. Each case was presented in a stepwise manner, with each step requiring students to submit answers to predefined questions and/or complete tasks (e.g., recommending a test, writing an assessment, performing an oral presentation, and calling a standardized patient with test results) in order to proceed to the next stage. Cases were written as typical admission or progress notes, so that students could become familiar with the standardized written language used in clinical documentation. Cases evolved over the course of 3 to 5 days, with increasing task complexity, mimicking situations that may occur in clinical settings; students often worked on 2 different cases each day, which simulated taking care of multiple patients. Fourth year student preceptors facilitated the sessions and were given detailed instructions on high-yield topics to cover but were not directly supervised during sessions. These peer mentors were similarly displaced and had limited opportunities for advanced electives and away rotations. Students submitted written assignments and recorded oral presentations onto the institution’s CANVAS learning management system (Instructure Inc., Salt Lake City, UT) and received feedback from faculty, senior students and assigned peer reviewers.

**Table 1.** Details of the nine cases and relevant EPAs covered

Clerkship	Chief Complaint	1^ary^ Diagnosis	EPA 1	EPA 2	EPA 3	EPA 4	EPA 5	EPA 6	EPA 7	EPA 10	Sessions	Communication Skills
Medicine	Dyspnea	Anemia	X	X	X	X	X	X	X	X	5	Specialty consultation
Primary Care	New patient visit	Hypertension	X	X	X	X	X	X	X		3	Telemedicine follow-up
Gynecology	Vaginal bleeding	Uterine cancer	X	X	X	X	X	X	X		3	Virtual follow-up
Pediatrics	Fever and cough	Cystic fibrosis	X	X	X	X	X	X	X		3	Family-centered rounds
Neurology	Seizure	Stroke	X	X	X	X	X	X	X	X	3	Specialty consultation
Obstetrics	Vaginal discharge	Premature labor	X	X	X	X	X	X	X	X	3	Oral presentation
Surgery	Abdominal pain	Cholecystitis	X	X	X	X			X		3	Oral examination
Psychiatry	Failure to thrive	Depression	X	X	X	X	X	X	X		3	Family collateral
Critical Care	Hypotension	Sepsis	X	X	X	X	X	X	X	X	3	Goals of care, consultation

Each clerkship was associated with 1 case. Association of American Medical Colleges (AAMC) Core Entrustable Professional Activities (EPAs). EPA 1 = Gather a History and Perform a Physical Examination, EPA 2 = Prioritize a Differential Diagnosis Following a Clinical Encounter, EPA 3 = Recommend and Interpret Common Diagnostic and Screening Tests, EPA 4 = Enter and Discuss Orders and Prescriptions, EPA 5 = Document a Clinical Encounter in the Patient Record, EPA 6 = Provide an Oral Presentation of a Clinical Encounter, EPA 7 = Form Clinical Questions and Retrieve Evidence to Advance Patient Care, EPA 10 = Recognize a Patient Requiring Urgent or Emergent Care and Initiate Evaluation and Management. Full description of each EPA is available at available at https://www.aamc.org/what-we-do/mission-areas/medical-education/cbme/core-epas/publications.

Six overarching principles guided the design of these cases: (1) we emphasized a constructivist philosophy [18], according to which students learn best by directly participating in the construction of their own knowledge. Thus, we adopted PBL as the primary teaching modality and encouraged value in the process of learning rather than getting the correct answers. (2) We focused on self-regulated learning, in which students could identify their strengths and gaps with constant feedback from their small group mentors and peers. Each step of a case required students to make predictions (e.g., formulate a differential diagnosis, recommend diagnostic testing, interpret laboratory results), which they then were able to check against the new information obtained. This allowed students to immediately reflect on their performance and calibrate their learning experience (Figure 1). (3) Students worked in small groups to encourage collaboration and team-based learning. (4) Each case focused on the teaching of authentic, high-yield clinical skills such as note-writing, oral presentations, calling a consultant, and communicating with patients and surrogates. Students were required to write and submit admission and/or progress notes for most of the cases as well as record themselves presenting the patient to the team. They also participated in two communication workshops with standardized patients. (5) The small groups were led by near-peer mentors (e.g., 4th year medical students) who were instructed to give students enough autonomy to work through the cases independently but were available to provide guidance and feedback. (6) A final principle driving our curricular design was Kolb’s experiential learning theory [19]; we wanted students to supplement learning from virtual encounters and small groups with authentic clinical experiences. Given the importance of reflection in Kolb’s learning cycle, each activity required a piece of reflective writing.

On an average day, students spent about 3 hours working through the cases in small groups, 2 hours completing assignments associated with each activity and 2 hours in traditional lectures, workshops or in the clinical space. Each student submitted and received feedback on 14 written assignments, including case write-ups, progress notes and follow-up visit notes. They performed 13 oral case presentations, completed 3 telemedicine standardized patient or family encounters and participated in one mock surgery oral exam. All students also attended a 4-hour communication workshop on delivering difficult news to patients.

### Course evaluation and statistical analysis

Students completed a questionnaire consisting of 20 questions which asked to rate self-perceived confidence in performing core clinical skills on a 5-point Likert scale (1 = not at all confident, 2 = slightly confident, 3 = somewhat confident, 4 = fairly confident and 5 = very confident). Questions are listed in Table 2. Survey response was not mandatory and did not affect the overall pass/fail grade in the course. Students were surveyed on day 1 (baseline), after week 2, and at the end of the course on week 4. Students were also surveyed 2 weeks after the beginning of their first clinical rotation to assess for sustained impact on self-efficacy. MD-PhD students (N = 18) were excluded because their entry into the clinical phase is normally scheduled 8 to 10 weeks after course completion. Week 2 and week 4 surveys were compared to the baseline survey. Post course surveys were compared to week 4 surveys. Statistical analyses were conducted using JMP Pro 14.0.0 (SAS Institute, Cary, NC). Descriptive statistics are reported. Paired t-tests were used to compare survey answers across timepoints. Results were considered statistically significant if P < 0.05.

**Table 2.** Pre and post-course self-efficacy ratings in performing core competencies

Core competencies & EPAs	Baseline	Week	Week 4	Post-Course
***EPA 1: Gather a History and Perform a Physical Examination***				
Q1. I can gather a patient’s history in a hypothesis-driven fashion.	2.65 ± 0.90	3.12 ± 0.90**	3.69 ± 0.77**	3.88 ± 0.57**^†^**
Q2. I can perform a physical examination on a patient.	2.35 ± 0.78	2.60 ± 0.95**	3.53 ± 0.84**	3.70 ± 0.80
Q10. I can effectively preround on a patient during a subsequent visit.	1.68 ± 0.79	2.61 ± 0.90**	3.24 ± 0.84**	3.68 ± 0.89^δ^
***EPA 2: Prioritize a Differential Diagnosis Following a Clinical Encounter***				
Q3. I have a systematic framework for approaching patient’s chief complaints.	2.27 ± 0.93	2.85 ± 0.83**	3.51 ± 0.74**	3.60 ± 0.66
Q4. I can use the history and physical examination to prioritize the differential diagnosis.	2.52 ± 0.93	3.17 ± 0.87**	3.69 ± 0.79**	3.70 ± 0.62
***EPA 3: Recommend and Interpret Common Diagnostic and Screening Tests***				
Q5. I can recommend laboratory tests and other diagnostic studies to help arrive at a diagnosis.	2.18 ± 0.83	2.97 ± 0.87**	3.55 ± 0.79**	3.58 ± 0.65
Q6. I can correctly interpret laboratory tests and diagnostic studies and use them to prioritize the differential diagnosis.	2.20 ± 0.78	3.04 ± 0.84**	3.48 ± 0.77**	3.64 ± 0.64
***EPA 4: Enter and Discuss Orders and Prescriptions***				
Q8. I can offer a diagnostic and therapeutic plan of management during a patient encounter.	1.70 ± 0.77	2.69 ± 0.81**	3.29 ± 0.78**	3.33 ± 0.84
***EPA 5: Document a Clinical Encounter in the Patient Record***				
Q7. I can document an interpretive summary and prioritized assessment of a patient encounter.	2.22 ± 0.94	3.09 ± 0.79**	3.64 ± 0.75**	3.77 ± 0.75
Q11. I can write a progress note or subsequent outpatient clinic note.	2.02 ± 0.88	3.19 ± 0.88**	3.69 ± 0.81**	3.77 ± 0.81
***EPA 6: Provide an Oral Presentation of a Clinical Encounter***				
Q9. I can provide an oral presentation of an initial patient encounter.	2.26 ± 0.97	2.92 ± 0.95**	3.63 ± 0.81**	3.79 ± 0.70
Q12. I can call a specialist team for a consultation.	1.37 ± 0.79	2.76 ± 0.91**	3.34 ± 0.82**	3.20 ± 0.92**^†^**
***EPA 7: Form Clinical Questions and Retrieve Evidence to Advance Patient Care***				
Q13. I can explain the plan of care to a patient after an encounter.	2.17 ± 1.04	3.05 ± 0.93**	3.56 ± 0.84**	3.69 ± 0.87
Q14. I can deliver bad news to a patient.	1.54 ± 0.91	2.24 ± 1.07**	3.31 ± 0.98**	3.13 ± 0.95^δ^
Q17. I can acknowledge my knowledge gaps and identify resources to fill them.	3.00 ± 0.88	3.75 ± 0.96**	4.11 ± 0.79**	4.24 ± 0.64
***EPA 9: Collaborate as a Member of an Interprofessional Team***				
Q15. I can work collaboratively with other health care professionals in inpatient and outpatient settings.	2.86 ± 1.12	3.43 ± 0.99**	4.09 ± 0.83**	4.21 ± 0.72
Q16. I can work effectively as a team member to solve problems and learn together.	3.28 ± 1.06	3.78 ± 0.90**	4.18 ± 0.78**	4.34 ± 0.67
***EPA 13: Identify System Failures and Contribute to a Culture of Safety and Improvement***				
Q18. I am able to receive feedback from others and use it as an opportunity for growth.	4.16 ± 0.83	4.24 ± 0.65	4.37 ± 0.67*	4.55 ± 0.52
Q19. I can give feedback to others in order to foster their growth.	3.41 ± 0.96	3.81 ± 0.79**	4.02 ± 0.83**	3.98 ± 0.75
***Other***				
Q20. I have strategies to maintain personal wellness that I can use in the coming year.	3.74 ± 0.86	3.92 ± 0.68*	4.04 ± 0.84**	3.93 ± 0.85

Likert scale of self-efficacy (1 = not at all confident, 2 = slightly confident, 3 = somewhat confident, 4 = fairly confident, 5 = very confident). Paired *t*-tests comparing weeks 2 and 4 versus baseline: *P < 0.05, **P < 0.01 (*N* ranged between 100–103) and comparing post-course versus week 4: **^†^**P < 0.05, ^δ^P < 0.01 (*N* ranged between 75 and 79). EPA = Entrustable Professional Activity, Q = question.

Content analysis was used to explore qualitative data. Additional open-ended questions were included to capture student sources of anxieties and confidence. Feedback on the value of in-person sessions including the physical diagnosis, OR shadowing and IPE swas also elicited. Examples of content identification and coding are shown in Supplementary Table S1.

## Results

The course was completed successfully by all 105 second year students enrolled. Overall, 19.5% of students rated the course as good, 44.2% as very good and 25.9% as excellent.

Prior to the half-day exercise in the OR, 64% of students had never participated in a procedure and 58% had never interacted with the anesthesia team. After the exercise, the percentage of students who reported feeling comfortable or very comfortable in the OR increased from 24% to 58%. Students agreed or strongly agreed that the IPE helped them develop greater respect for other health professionals (95%), learn how to engage with others to meet patient care needs (93%), and work effectively in teams (97%). Finally, shadowing an advanced clerkship student on pre-rounds/rounds made 86% of students feel more comfortable in the physical space of a hospital ward.

### Improvement in self-efficacy

Self-efficacy survey results are reported in Table 2. At baseline, students were somewhat to fairly confident in several attitudinal domains, such as their ability to work effectively as a team member, recognize knowledge gaps, receive and provide feedback and maintain wellness. However, most were less confident in their clinical skills. By the fourth week of the course, students’ self-reported efficacy in carrying out these tasks increased significantly. The greatest improvements were observed in students’ perceived confidence in calling a consult (1.37 vs. 3.20, P < 0.01), writing a progress note (2.02 vs. 3.77, P < 0.01), offering a diagnostic and therapeutic plan of management (1.70 vs. 3.33, P < 0.01), and pre-rounding (1.68 vs. 3.68, P < 0.01).

Among 87 eligible students who entered the clinical curriculum during our study, 79 (91%) completed the post-course survey. Two weeks into their first clerkships, students had levels of confidence which were not significantly different from those at the end of the course, suggesting a sustained impact (Table 2). Notable exceptions were pre-rounding and calling a consult for which students felt less confident after beginning clerkships.

### Self-reported anxieties and confidence with transition

**Figure 2.**
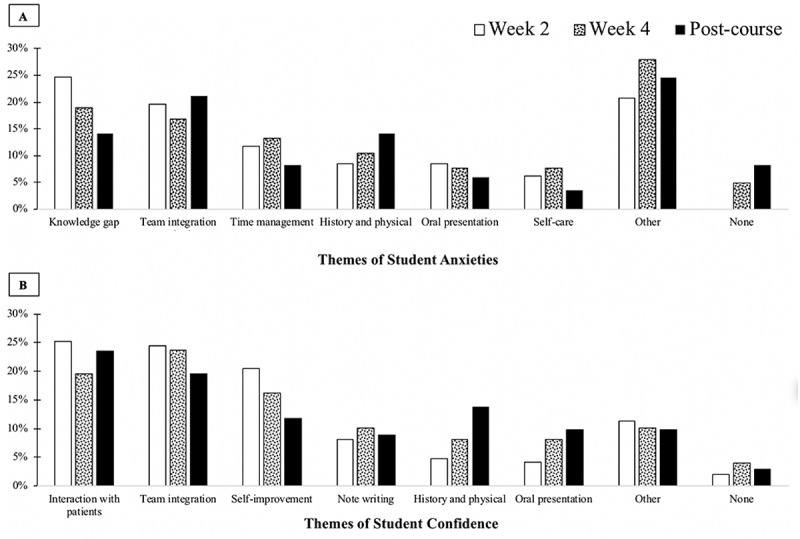
Panel A reports content analysis of responses to: *‘What are you most anxious about as you prepare to begin (or now that you are in) your first clerkship.’* Other included adjustment to the new environment, procedural skills, navigating the electronic medical record, grading, etc. Panel B reports content analysis of responses to: *‘What are you most confident about as you prepare to begin (or now that you are in) your first clerkship.’* Other included wellness, general clinical skills and time management. Frequencies indicate % of theme/total number of phrases

Students were asked about anxieties related to their transition to the clinical phase of their curriculum at different timepoints (Figure 2(a); Supplementary Table S1). The sources of anxiety most frequently mentioned were perceived knowledge gaps, integrating into teams, navigating their new role, and managing time. Anxieties about the ability to perform clinical skills persisted throughout the course. Concerns about COVID-19 were rarely mentioned.

Students were also asked about what areas they felt most confident in (Figure 2(b); Supplementary Table S1). Most reported feeling confident in their ability to interact with patients and build relationships with their team. Several students expressed a growth mindset and a willingness to make mistakes.

## Discussion

The COVID-19 pandemic caused considerable disruption in medical education. In the U.S., most institutions pulled students out of the clinical environment to ensure student and patient safety and safeguard scarce resources [14,20]. However, the pandemic also served as a catalyst for several curricular innovations designed to circumvent these challenges [14,21]. Virtual electives were implemented at several medical schools: students helped with COVID-focused literature reviews for clinicians [10], participated in telehealth-based patient care [16], enrolled in courses anchored on workplace learning as a substitute for clinical clerkships [11], and participated in several clerkship-specific initiatives aimed at providing remote instruction [12,13,15].

These initiatives were successful at addressing specific challenges during the pandemic, but adequate solutions to the broader problem of effectively transitioning preclinical students into the clinical phase of the curriculum have not been specifically reported in the literature. The pandemic, combined with preexisting widespread inadequacies in clerkship preparedness, made it even more challenging for preclinical students to hone their clinical skills via in-person interactions with patients, simulations, and skill building sessions.

Pre-COVID-19, various TTC initiatives had been developed at medical institutions around the country; yet they vary considerably in structure and content, and some of the highest yield topics for day-to-day life on the wards are often not covered [1,22,23]. O’Brien et al., for example, surveyed 83 medical schools, among which 73 reported having some form of transitional course, but only 14 of such courses included experiences in the clinical setting [1]. Less than a third of the schools surveyed covered how to properly do a history and physical, interpret laboratory data or use basic medical equipment and only about 50% of the courses tackled note-writing, order entry and oral presentations [1]. It is likely that many institutions have since innovated their TTC curricula; however, no comprehensive surveys of the current landscape have been detailed in the medical education literature.

We chose to use COVID-19 disruptions as a starting point for a broader re-thinking and restructuring of the TTC phase. This novel course served as a bridge between the classroom and the wards. We developed meaningful, skill-building clinical experiences in a supervised setting making sure content was carefully anchored to key EPAs [17], and was carefully selected through an iterative approach that involved all stakeholders (e.g., preclinical students, clinical students, and clerkship directors). Instruction combined a series of core teaching principles and a multimodal approach which have not been used to this extent in prior TTC courses. We also surveyed students at multiple time-points, relying on responses to make real-time changes to the course. When surveyed, all students reported significant improvements compared to baseline in the key competencies necessary to perform adequately on clerkship. The granularity of our questionnaire allowed us to pinpoint specific areas of improvement and/or weakness. Improvements in self-efficacy were sustained even after students began their first rotation.

Activities encouraged students to learn in a self-directed, team-based fashion, focusing on enhancing clinical reasoning skills. We relied on near-peer 4^th^-year student mentors for day-to-day instruction. A growing body of literature have described the benefits of utilizing near-peer mentors in medical education [24,25] and as teachers in prior TTC initiatives [26]. Prior to the COVID-19 pandemic, recruiting a large cohort of 4^th^-year students would have been challenging. This course offered students interested in medical education the direct, structured, and intense teaching experiences in lieu of disrupted clinical rotations.

Students applied knowledge gained during small group sessions to real patient encounters in the hospital, and/or standardized patients virtually. In our course, students spent 2 sessions working alongside nurses and operating room personnel to engender the interprofessionalism attitudes that are helpful for later clinical success. Although different models of interprofessional education have been described [27] most preclinical students have few opportunities to interact with other healthcare professionals. During our course, strict limitations on the presence of learners in the clinical space persisted, but the students’ expressed appreciation of these exercises suggest that future iterations of the course could include more sessions and also with other healthcare professionals such as, for example, advanced practice providers (e.g., nurse practitioners and physician assistants), physical therapists and social workers.

There were several limitations to this study. First, the self-reported nature of our data allows room for bias, especially towards the conclusion of the course. However, results from content analysis of students’ narrative responses corroborated the quantitative scoring of self-efficacy. Second, our follow-up period was relatively short. However, self-efficacy would be expected to increase during clerkship year from educational experiences distinct from our intervention. Third, we lacked a more objective method to assess course efficacy and post-course improvements. We considered comparing clerkship evaluation scores and/or National Board Medical Examiners (NBME) subject exam scores to previous classes who did not complete this course, but we reasoned that such approach would be problematic in and of itself given neither would holistically capture the intricacy of overall clerkship preparedness, a central goal of the course. In addition, there was a considerable restructuring in the clinical curriculum in response to COVID-19; grading was switched to pass/fail and some rotations were shortened from 4 to 6 weeks. This made it impossible to compare our cohort with prior years. Self-efficacy measures are inherently subjective; however, to our knowledge, there has not been an externally validated scoring system to capture the same data type. Self-efficacy is recognized as the primary driver of motivation [28], and there are known correlations between perceived self-efficacy and academic performance [29,30]. Finally, a perceived lack of homogenization in teaching across groups may be viewed by some as a limitation of this course. However, we emphasized a constructivist philosophy and hoped to engender self-directed learning and foster individualization of learning goals. This mirrors the experiential learning during clerkships, where students must actively construct their own learning objectives based on their rotation, patients’, and the team’s daily needs. For many students, this is a challenge during the transition to clerkship.

This was a well-received, novel course that helped students prepare for their first clinical rotation. Despite many COVID-related restrictions on students being lifted, the pandemic’s residual effects (e.g., changes in budgets for standardized patient encounters and safety requirements for interaction with patients) may continue to impact students’ in-person experiences in the preclinical phase in the coming years. The inherent flexibility of our course design is crucial in continuing to circumvent these restrictions while cultivating clinical skills. Given the success of the course, we believe that its core tenets are generalizable, even in the absence of restrictions, and could be implemented at other institutions. Our emphasis on self-directed learning and near-peer mentors, while anchoring skill building to core EPAs could be employed throughout the preclinical curriculum to more systematically train students.
